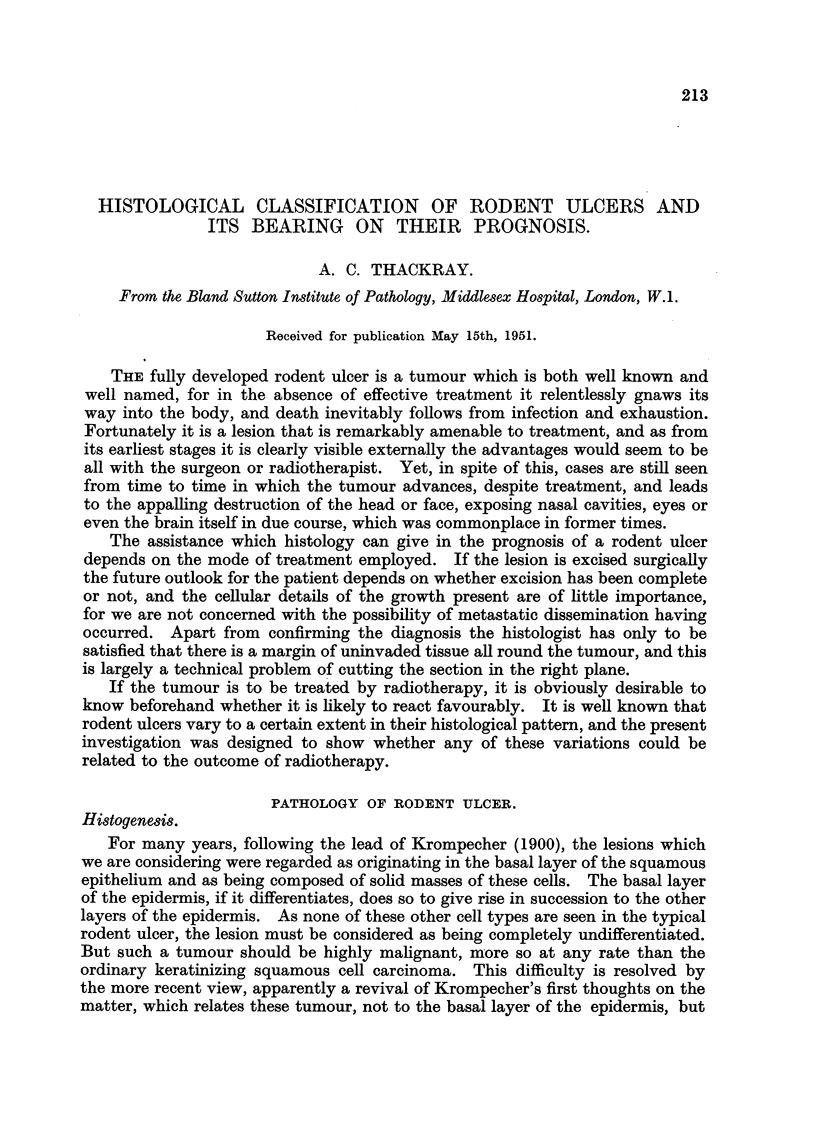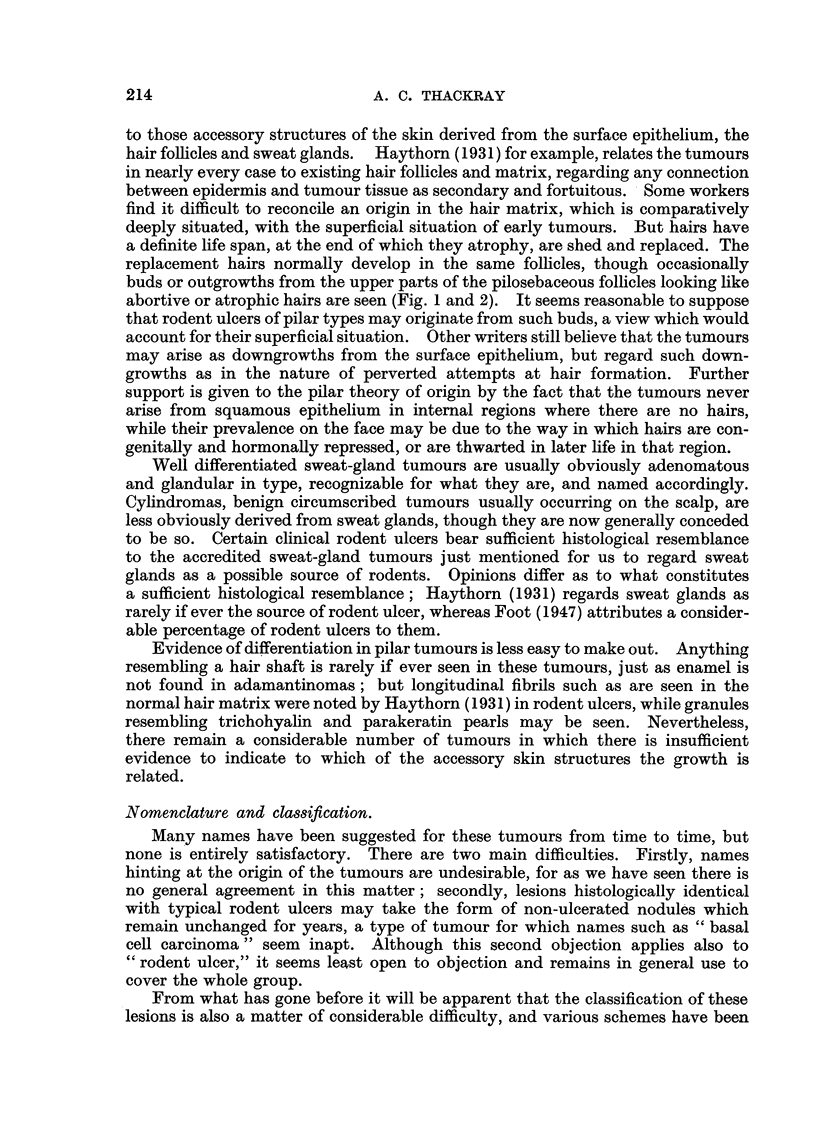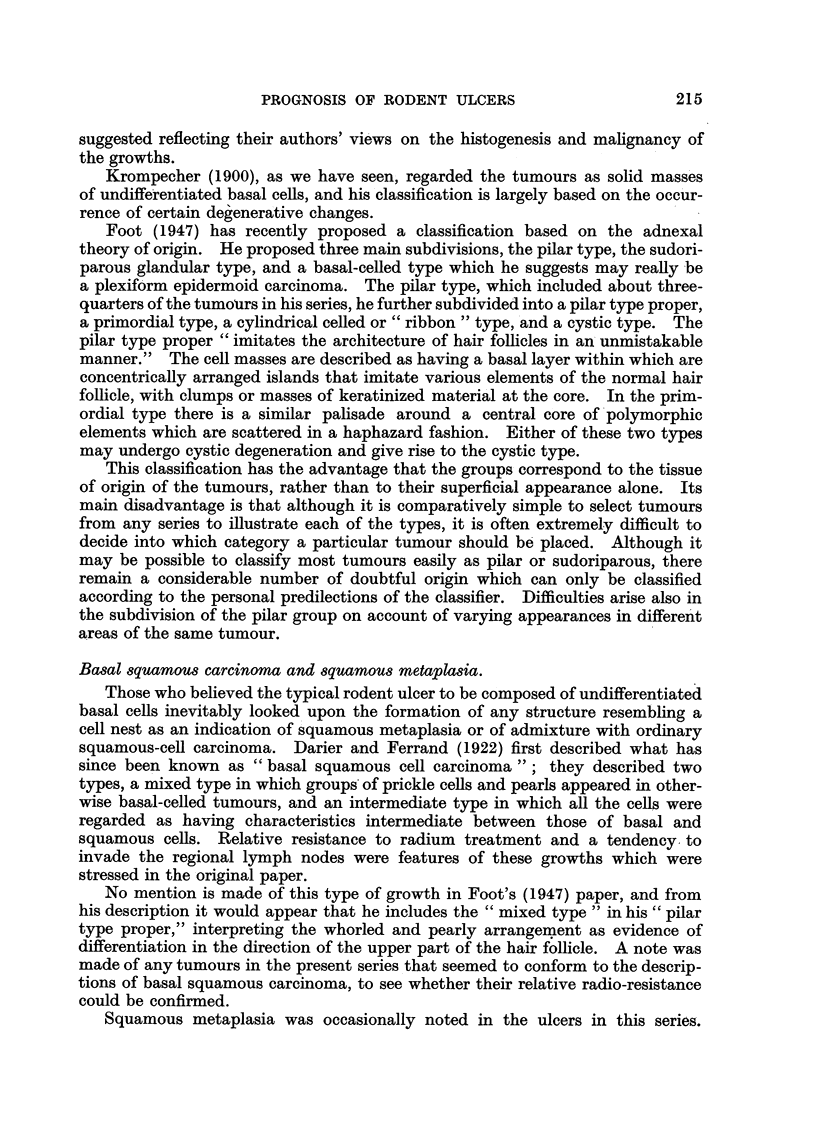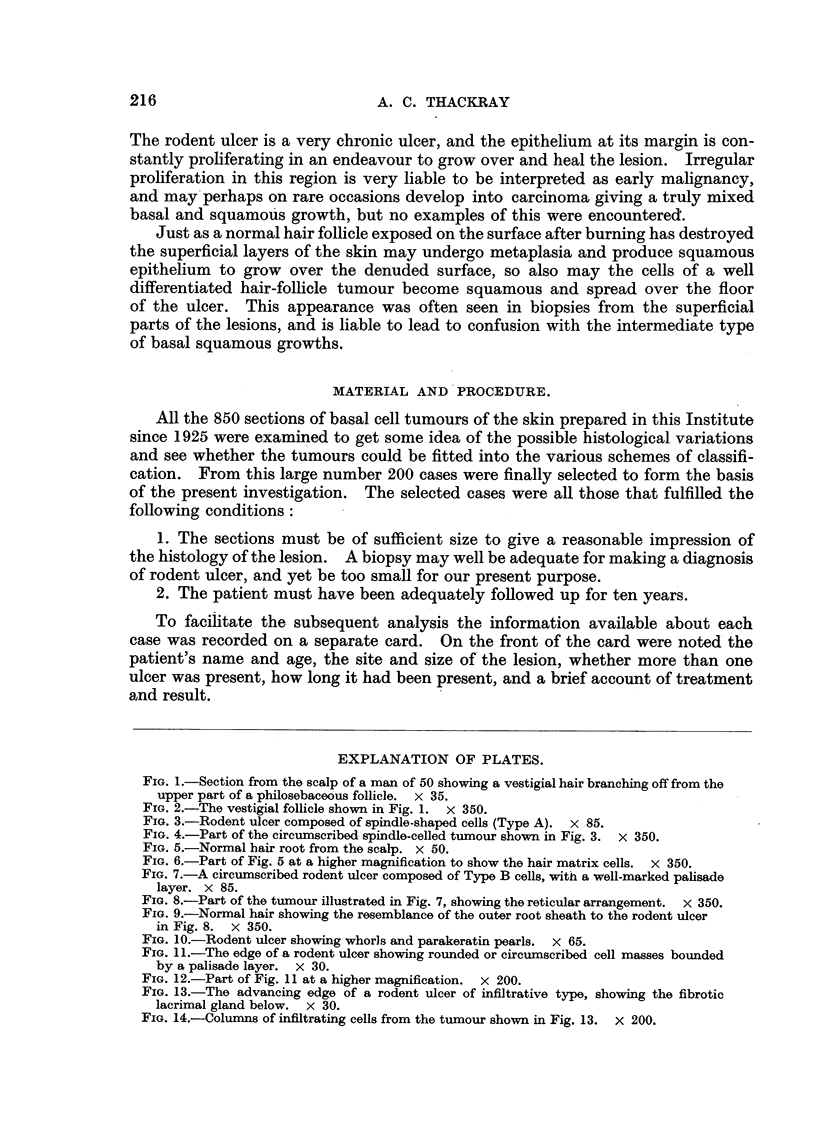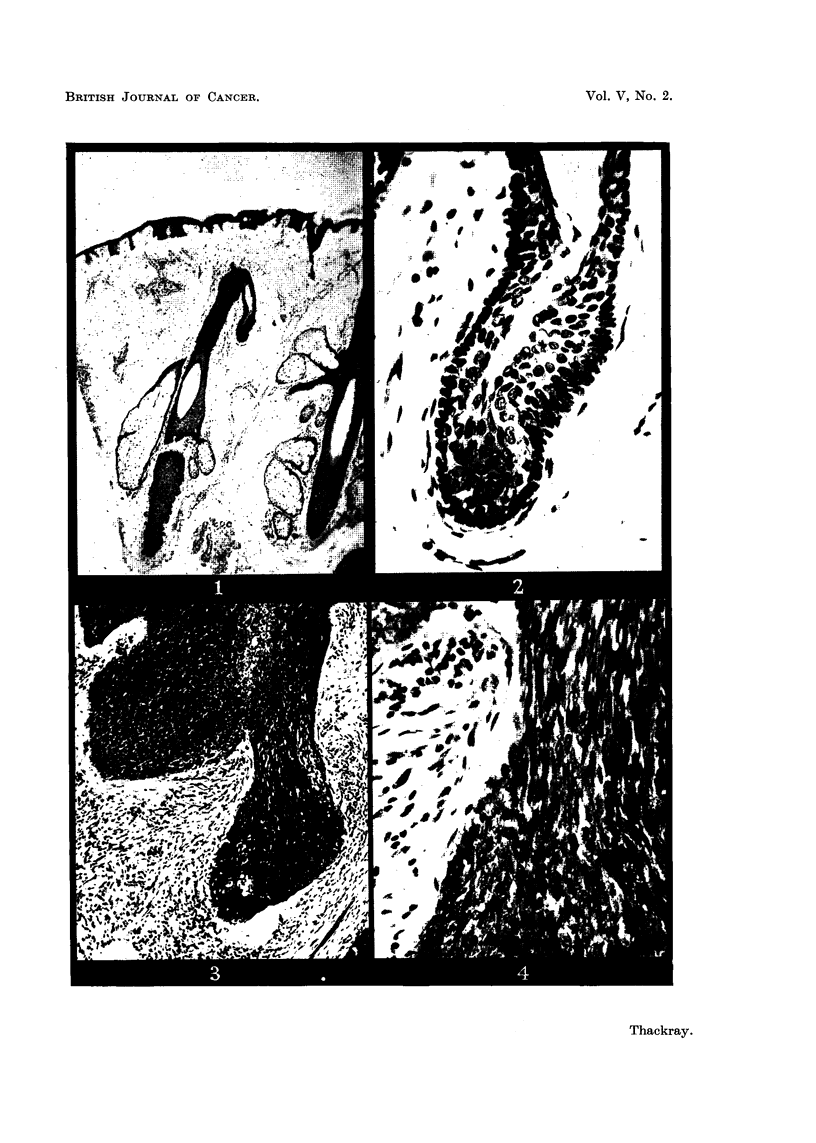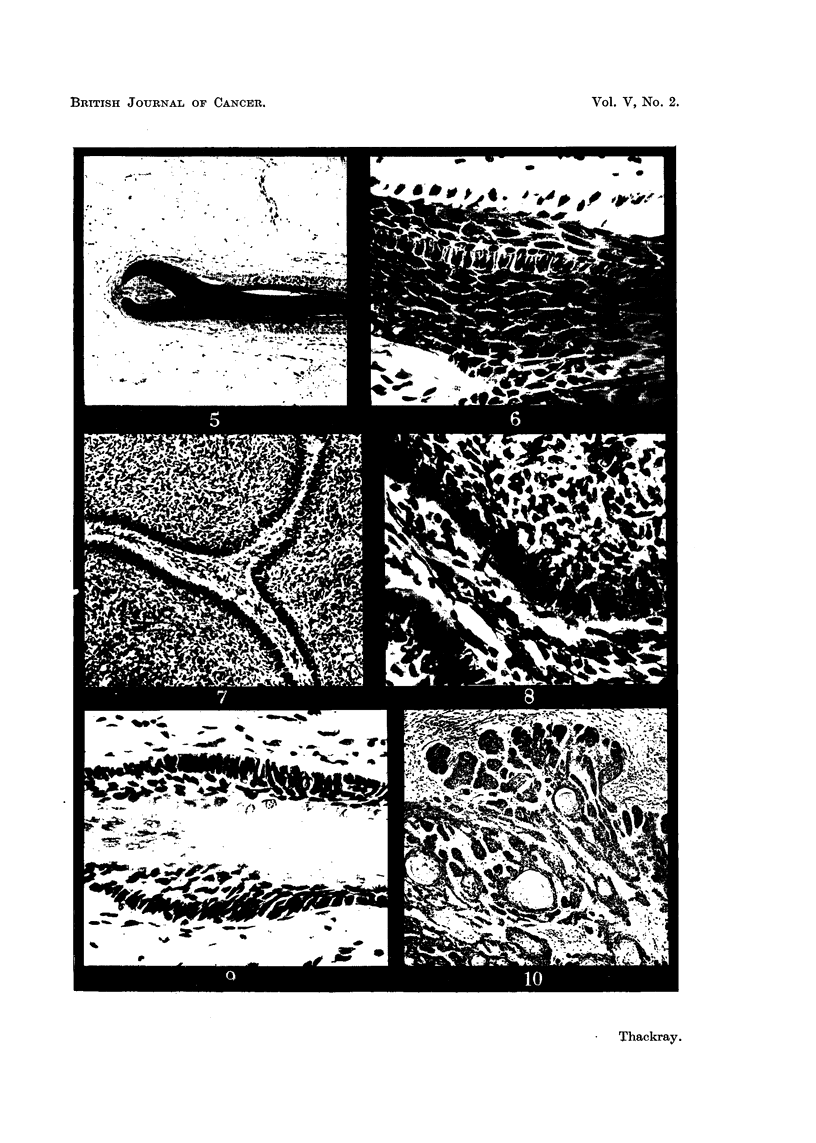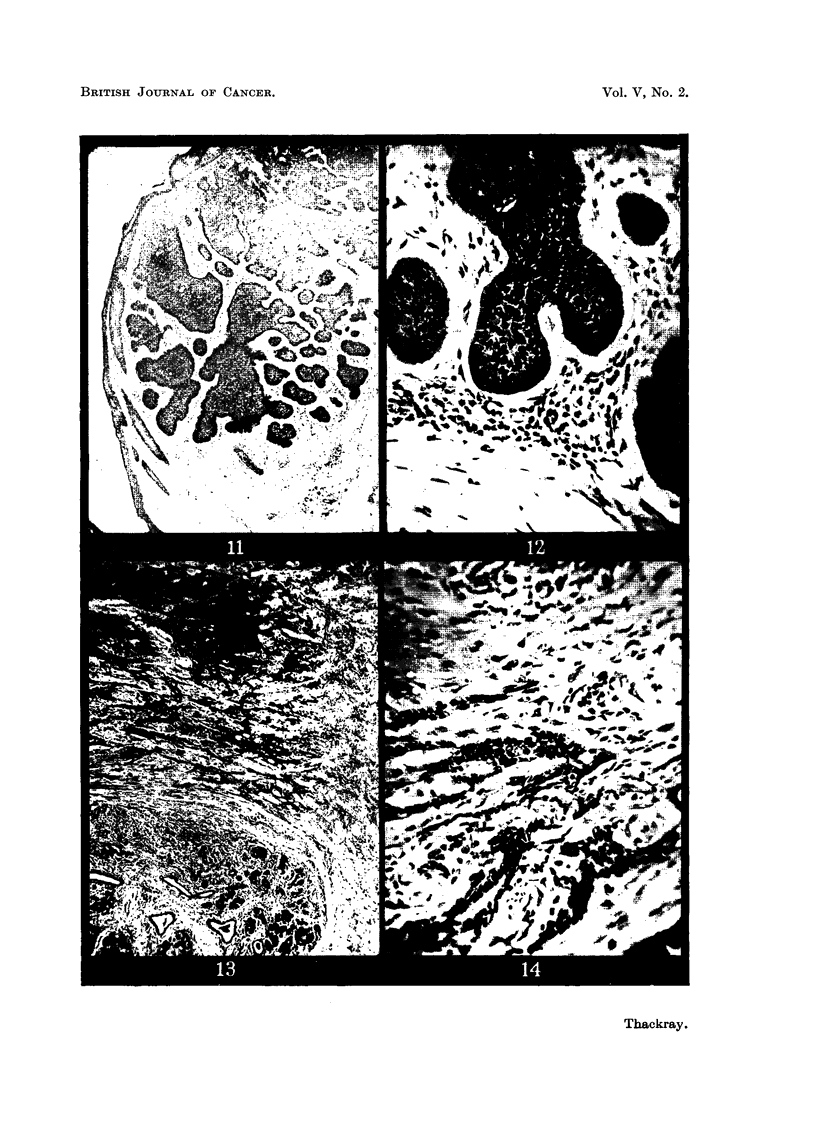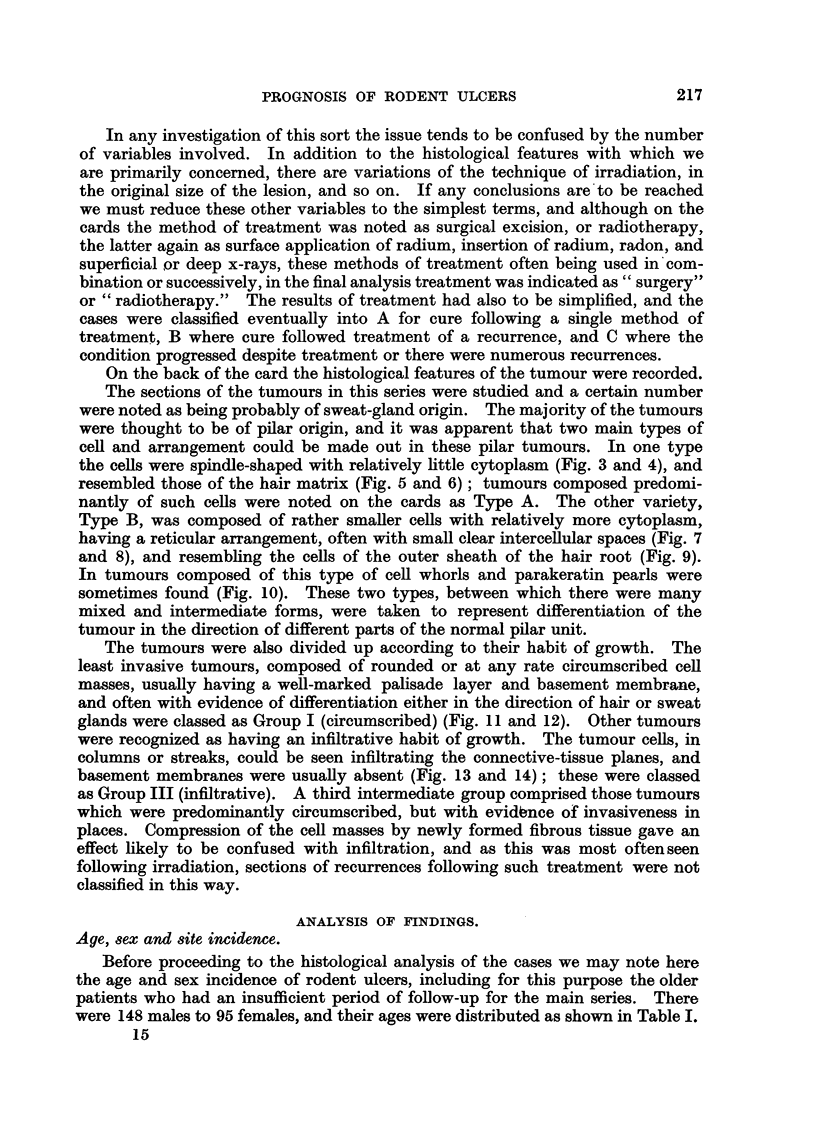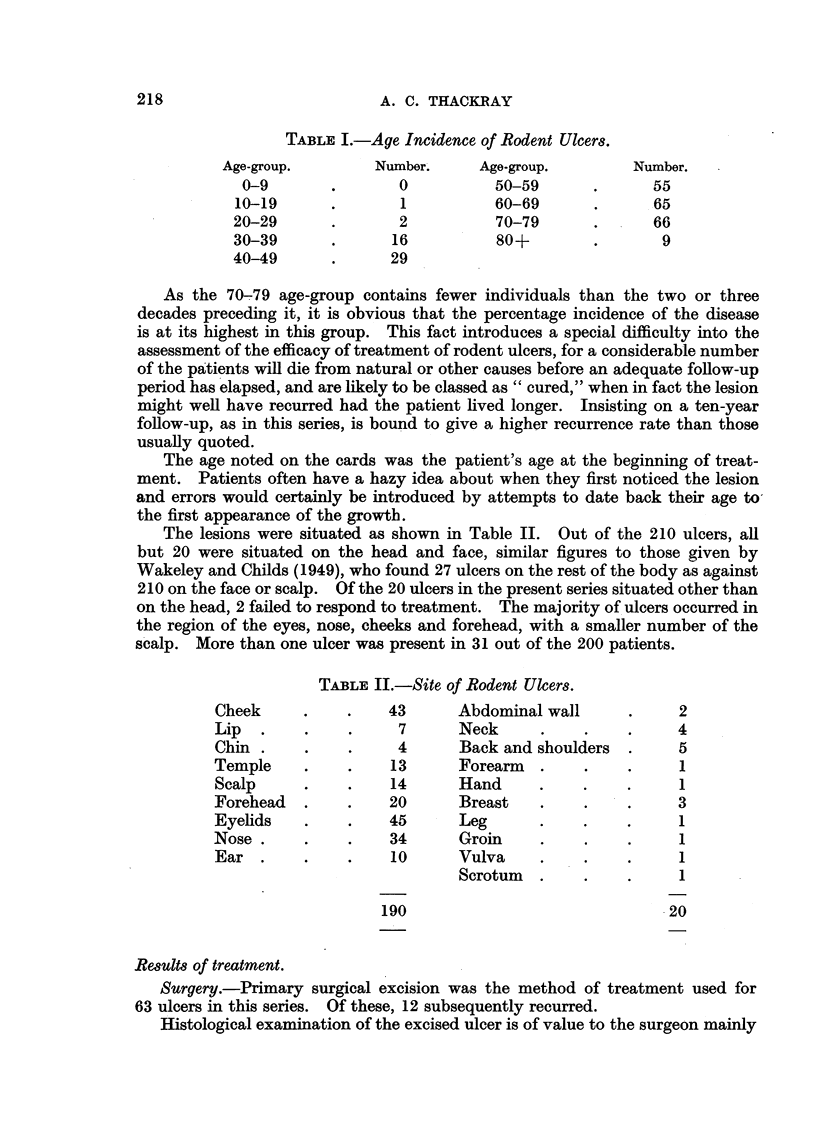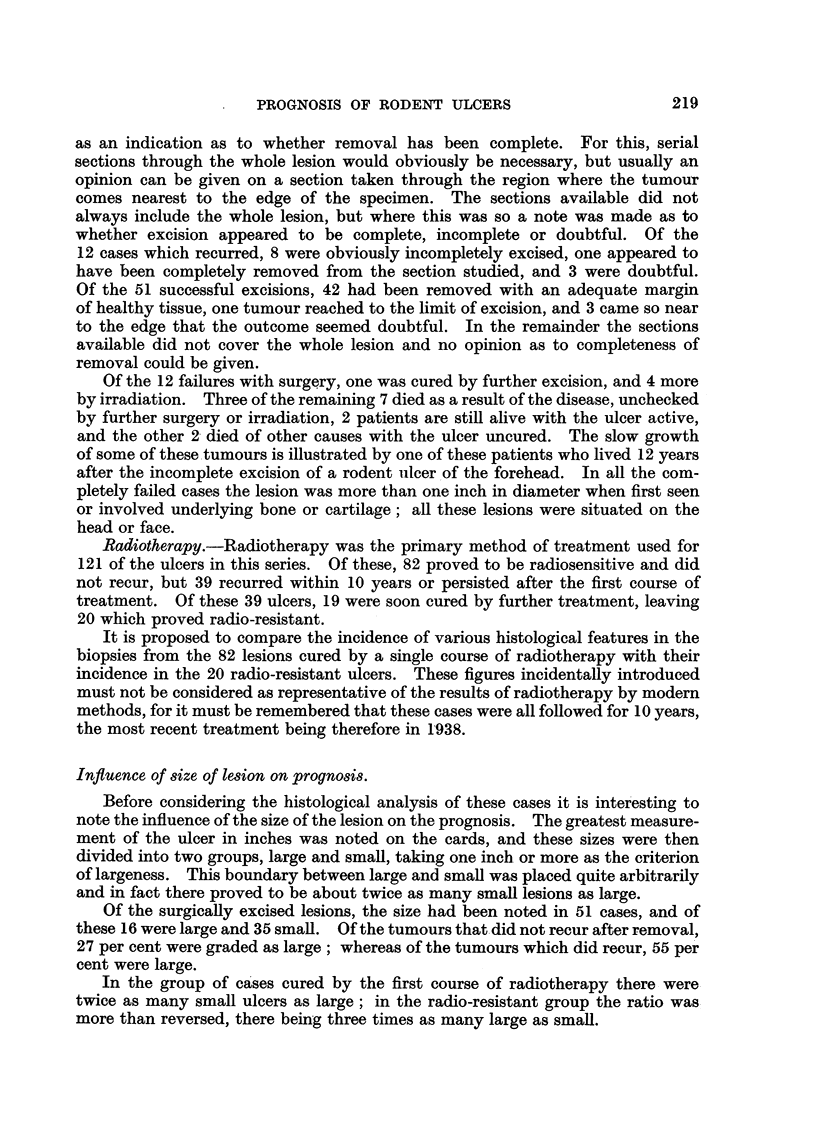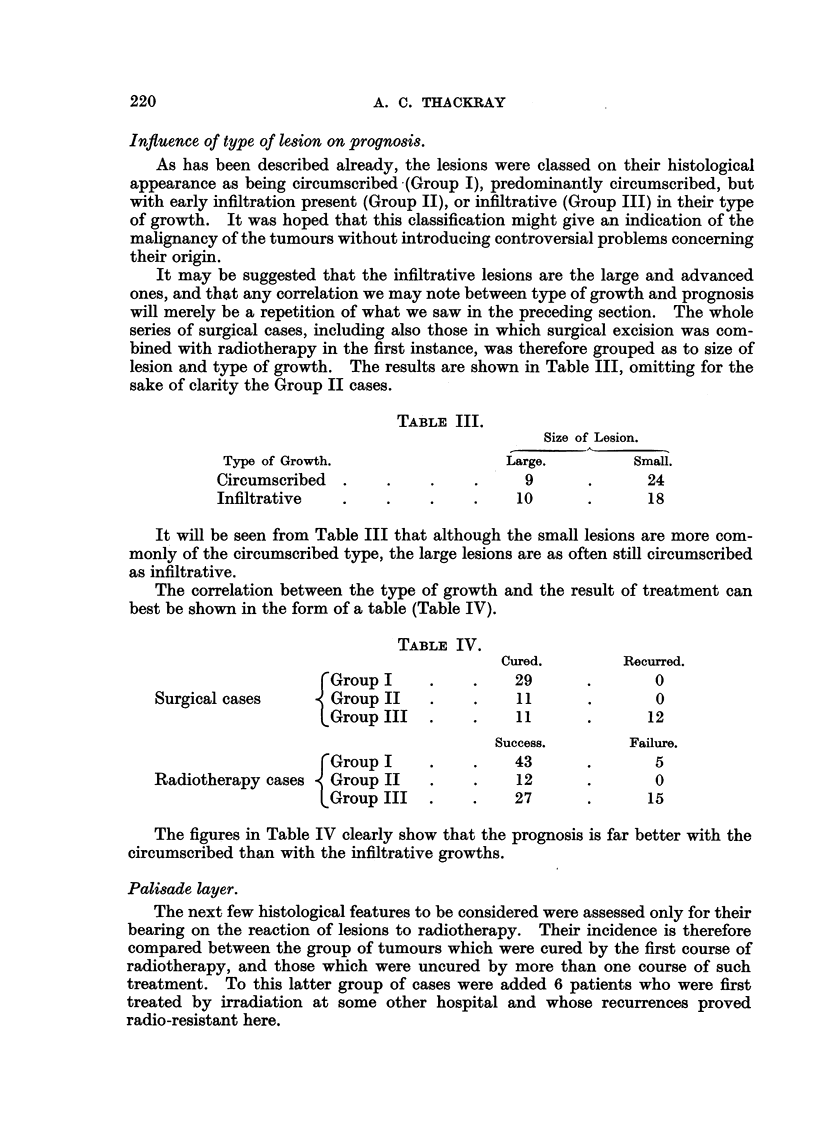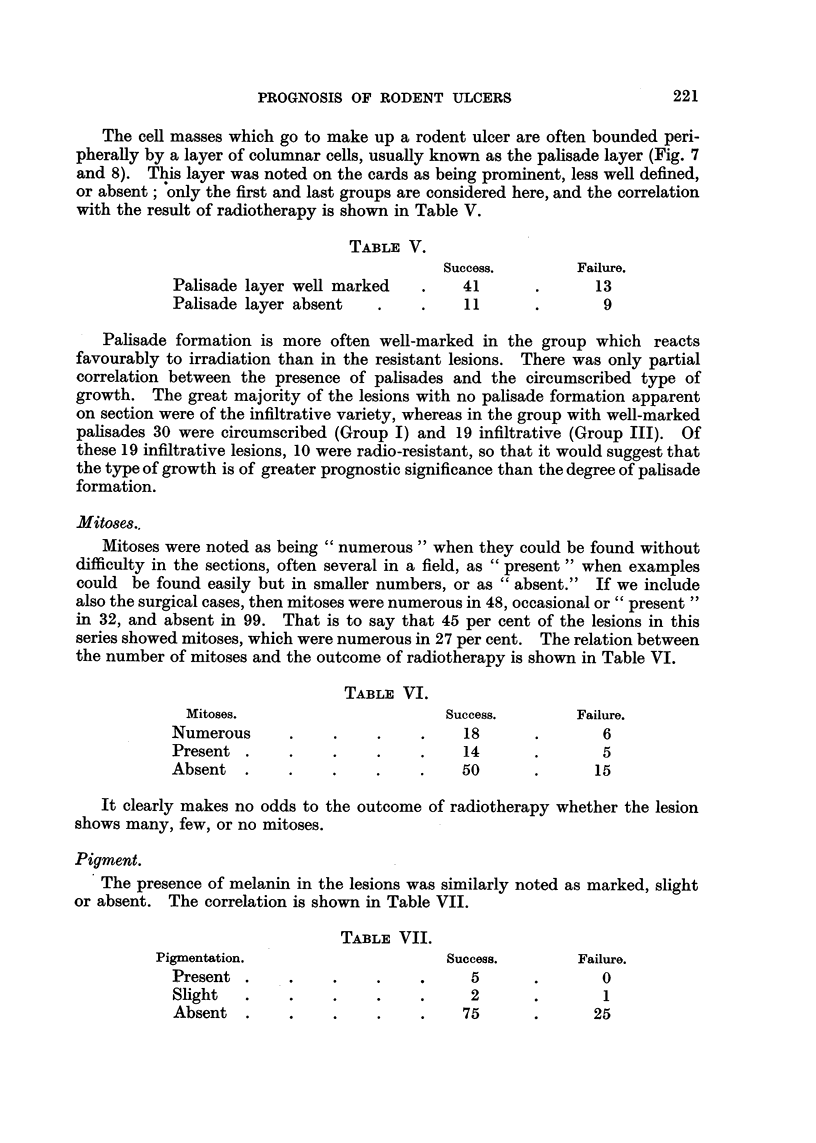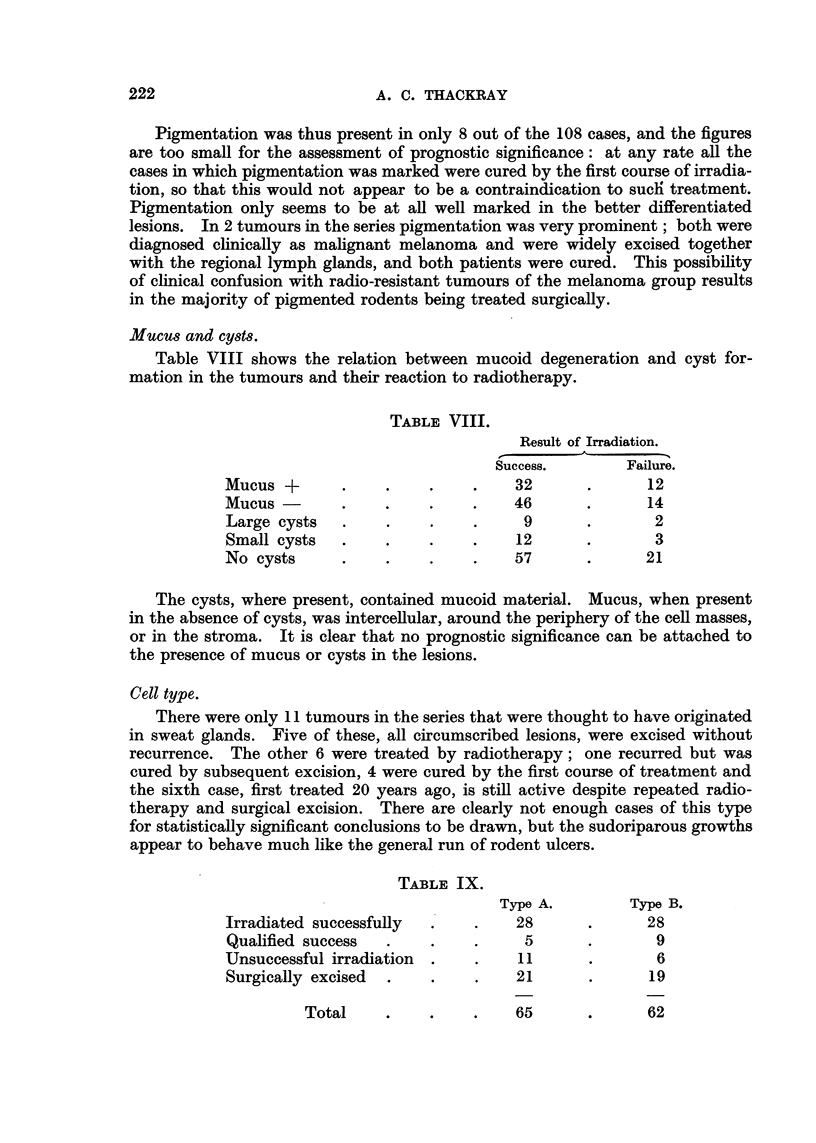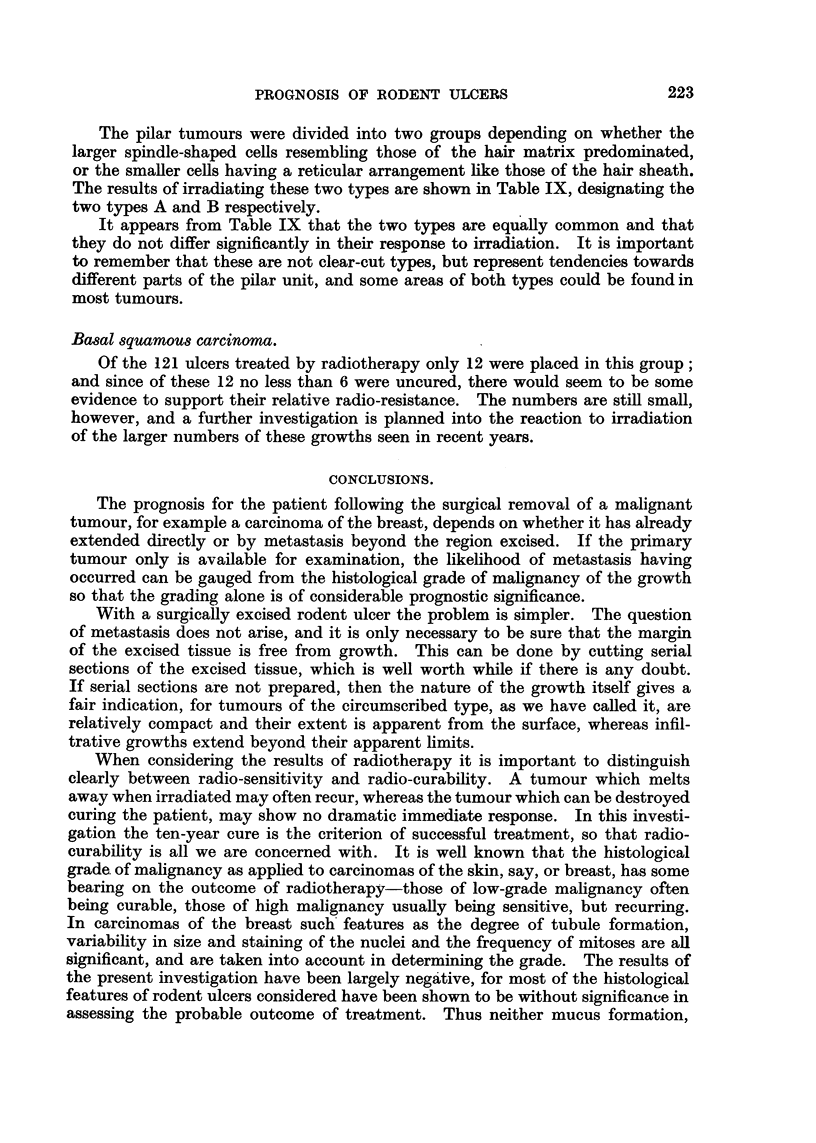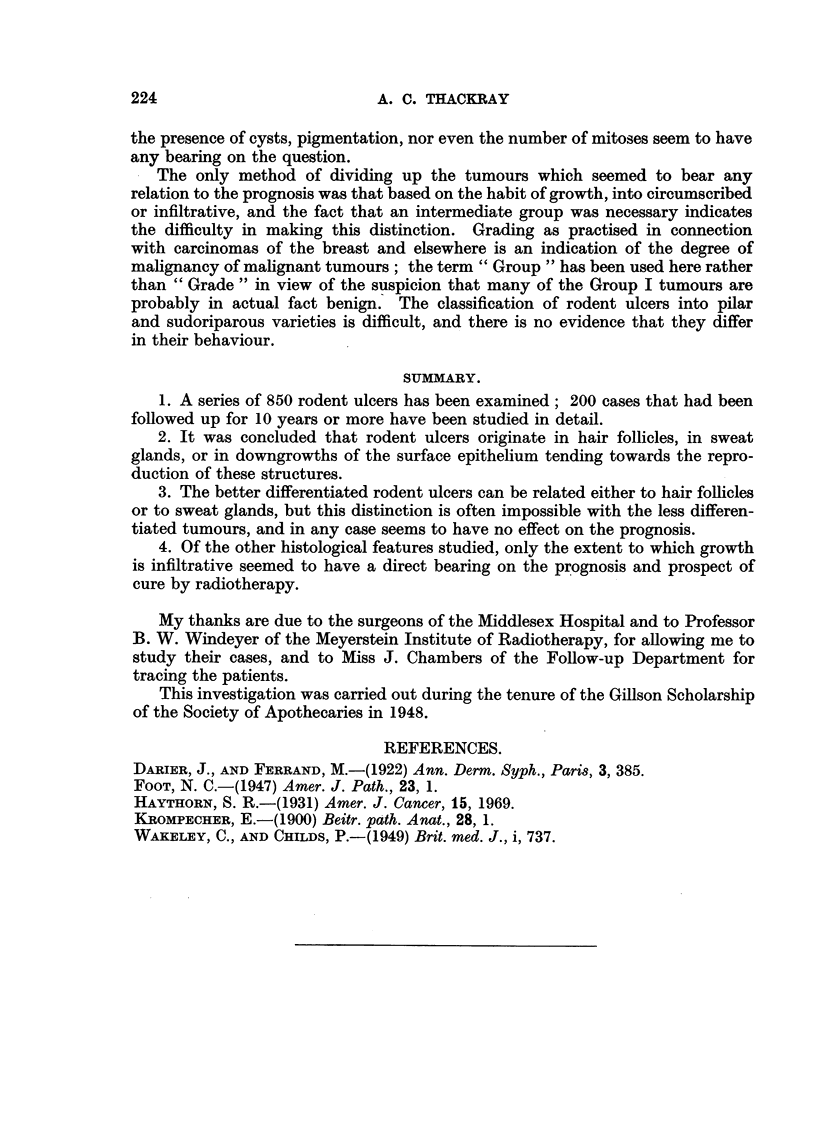# Histological Classification of Rodent Ulcers and its Bearing on their Prognosis

**DOI:** 10.1038/bjc.1951.22

**Published:** 1951-06

**Authors:** A. C. Thackray

## Abstract

**Images:**


### HISTOLOGICAL CLASSIFICATION OF RODENT ULCERS AND ITS BEARING ON THEIR PROGNOSIS.

A. C. THACKRAY.

*From the Bland Sutton Institute of Pathology, Middlesex Hospital, London, W.1.*

Received for publication May 15th, 1951.

THE fully developed rodent ulcer is a tumour which is both well known and well named, for in the absence of effective treatment it relentlessly gnaws its way into the body, and death inevitably follows from infection and exhaustion. Fortunately it is a lesion that is remarkably amenable to treatment, and as from its earliest stages it is clearly visible externally the advantages would seem to be all with the surgeon or radiotherapist. Yet, in spite of this, cases are still seen from time to time in which the tumour advances, despite treatment, and leads to the appalling destruction of the head or face, exposing nasal cavities, eyes or even the brain itself in due course, which was commonplace in former times.

The assistance which histology can give in the prognosis of a rodent ulcer depends on the mode of treatment employed. If the lesion is excised surgically the future outlook for the patient depends on whether excision has been complete or not, and the cellular details of the growth present are of little importance, for we are not concerned with the possibility of metastatic dissemination having occurred. Apart from confirming the diagnosis the histologist has only to be satisfied that there is a margin of uninvaded tissue all round the tumour, and this is largely a technical problem of cutting the section in the right plane.

If the tumour is to be treated by radiotherapy, it is obviously desirable to know beforehand whether it is likely to react favourably. It is well known that rodent ulcers vary to a certain extent in their histological pattern, and the present investigation was designed to show whether any of these variations could be related to the outcome of radiotherapy.

### PATHOLOGY OF RODENT ULCER.

### *Histogenesis.*

For many years, following the lead of Krompecher (1900), the lesions which we are considering were regarded as originating in the basal layer of the squamous epithelium and as being composed of solid masses of these cells. The basal layer of the epidermis, if it differentiates, does so to give rise in succession to the other layers of the epidermis. As none of these other cell types are seen in the typical rodent ulcer, the lesion must be considered as being completely undifferentiated. But such a tumour should be highly malignant, more so at any rate than the ordinary keratinizing squamous cell carcinoma. This difficulty is resolved by the more recent view, apparently a revival of Krompecher's first thoughts on the matter, which relates these tumour, not to the basal layer of the epidermis, but to those accessory structures of the skin derived from the surface epithelium, the hair follicles and sweat glands. Haythorn (1931) for example, relates the tumours in nearly every case to existing hair follicles and matrix, regarding any connection between epidermis and tumour tissue as secondary and fortuitous. Some workers find it difficult to reconcile an origin in the hair matrix, which is comparatively deeply situated, with the superficial situation of early tumours. But hairs have a definite life span, at the end of which they atrophy, are shed and replaced. The replacement hairs normally develop in the same follicles, though occasionally buds or outgrowths from the upper parts of the pilosebaceous follicles looking like abortive or atrophic hairs are seen (Fig. 1 and 2). It seems reasonable to suppose that rodent ulcers of pilar types may originate from such buds, a view which would account for their superficial situation. Other writers still believe that the tumours may arise as downgrowths from the surface epithelium, but regard such downgrowths as in the nature of perverted attempts at hair formation. Further support is given to the pilar theory of origin by the fact that the tumours never arise from squamous epithelium in internal regions where there are no hairs, while their prevalence on the face may be due to the way in which hairs are congenitally and hormonally repressed, or are thwarted in later life in that region.

Well differentiated sweat-gland tumours are usually obviously adenomatous and glandular in type, recognizable for what they are, and named accordingly. Cylindromas, benign circumscribed tumours usually occurring on the scalp, are less obviously derived from sweat glands, though they are now generally conceded to be so. Certain clinical rodent ulcers bear sufficient histological resemblance to the accredited sweat-gland tumours just mentioned for us to regard sweat glands as a possible source of rodents. Opinions differ as to what constitutes a sufficient histological resemblance; Haythorn (1931) regards sweat glands as rarely if ever the source of rodent ulcer, whereas Foot (1947) attributes a considerable percentage of rodent ulcers to them.

Evidence of differentiation in pilar tumours is less easy to make out. Anything resembling a hair shaft is rarely if ever seen in these tumours, just as enamel is not found in adamantinomas; but longitudinal fibrils such as are seen in the normal hair matrix were noted by Haythorn (1931) in rodent ulcers, while granules resembling trichohyalin and parakeratin pearls may be seen. Nevertheless, there remain a considerable number of tumours in which there is insufficient evidence to indicate to which of the accessory skin structures the growth is related.

*Nomenclature and classification.*

Many names have been suggested for these tumours from time to time, but none is entirely satisfactory. There are two main difficulties. Firstly, names hinting at the origin of the tumours are undesirable, for as we have seen there is no general agreement in this matter; secondly, lesions histologically identical with typical rodent ulcers may take the form of non-ulcerated nodules which remain unchanged for years, a type of tumour for which names such as "basal cell carcinoma" seem inapt. Although this second objection applies also to "rodent ulcer," it seems least open to objection and remains in general use to cover the whole group.

From what has gone before it will be apparent that the classification of these lesions is also a matter of considerable difficulty, and various schemes have been suggested reflecting their authors' views on the histogenesis and malignancy of the growths.

Krompecher (1900), as we have seen, regarded the tumours as solid masses of undifferentiated basal cells, and his classification is largely based on the occurrence of certain degenerative changes.

Foot (1947) has recently proposed a classification based on the adnexal theory of origin. He proposed three main subdivisions, the pilar type, the sudoriparous glandular type, and a basal-celled type which he suggests may really be a plexiform epidermoid carcinoma. The pilar type, which included about three-quarters of the tumours in his series, he further subdivided into a pilar type proper, a primordial type, a cylindrical celled or " ribbon " type, and a cystic type. The pilar type proper " imitates the architecture of hair follicles in an unmistakable manner." The cell masses are described as having a basal layer within which are concentrically arranged islands that imitate various elements of the normal hair follicle, with clumps or masses of keratinized material at the core. In the primordial type there is a similar palisade around a central core of polymorphic elements which are scattered in a haphazard fashion. Either of these two types may undergo cystic degeneration and give rise to the cystic type.

This classification has the advantage that the groups correspond to the tissue of origin of the tumours, rather than to their superficial appearance alone. Its main disadvantage is that although it is comparatively simple to select tumours from any series to illustrate each of the types, it is often extremely difficult to decide into which category a particular tumour should be placed. Although it may be possible to classify most tumours easily as pilar or sudoriparous, there remain a considerable number of doubtful origin which can only be classified according to the personal predilections of the classifier. Difficulties arise also in the subdivision of the pilar group on account of varying appearances in different areas of the same tumour.

*Basal squamous carcinoma and squamous metaplasia.*

Those who believed the typical rodent ulcer to be composed of undifferentiated basal cells inevitably looked upon the formation of any structure resembling a cell nest as an indication of squamous metaplasia or of admixture with ordinary squamous-cell carcinoma. Darier and Ferrand (1922) first described what has since been known as " basal squamous cell carcinoma " ; they described two types, a mixed type in which groups of prickle cells and pearls appeared in otherwise basal-celled tumours, and an intermediate type in which all the cells were regarded as having characteristics intermediate between those of basal and squamous cells. Relative resistance to radium treatment and a tendency to invade the regional lymph nodes were features of these growths which were stressed in the original paper.

No mention is made of this type of growth in Foot's (1947) paper, and from his description it would appear that he includes the " mixed type " in his " pilar type proper," interpreting the whorled and pearly arrangement as evidence of differentiation in the direction of the upper part of the hair follicle. A note was made of any tumours in the present series that seemed to conform to the descriptions of basal squamous carcinoma, to see whether their relative radio-resistance could be confirmed.

Squamous metaplasia was occasionally noted in the ulcers in this series.

The rodent ulcer is a very chronic ulcer, and the epithelium at its margin is constantly proliferating in an endeavour to grow over and heal the lesion. Irregular proliferation in this region is very liable to be interpreted as early malignancy, and may perhaps on rare occasions develop into carcinoma giving a truly mixed basal and squamous growth, but no examples of this were encountered.

Just as a normal hair follicle exposed on the surface after burning has destroyed the superficial layers of the skin may undergo metaplasia and produce squamous epithelium to grow over the denuded surface, so also may the cells of a well differentiated hair-follicle tumour become squamous and spread over the floor of the ulcer. This appearance was often seen in biopsies from the superficial parts of the lesions, and is liable to lead to confusion with the intermediate type of basal squamous growths.

### MATERIAL AND PROCEDURE.

All the 850 sections of basal cell tumours of the skin prepared in this Institute since 1925 were examined to get some idea of the possible histological variations and see whether the tumours could be fitted into the various schemes of classification. From this large number 200 cases were finally selected to form the basis of the present investigation. The selected cases were all those that fulfilled the following conditions :

1. The sections must be of sufficient size to give a reasonable impression of the histology of the lesion. A biopsy may well be adequate for making a diagnosis of rodent ulcer, and yet be too small for our present purpose.
2. The patient must have been adequately followed up for ten years.

To facilitate the subsequent analysis the information available about each case was recorded on a separate card. On the front of the card were noted the patient's name and age, the site and size of the lesion, whether more than one ulcer was present, how long it had been present, and a brief account of treatment and result.

---

### EXPLANATION OF PLATES.

FIG. 1.—Section from the scalp of a man of 50 showing a vestigial hair branching off from the upper part of a philosebaceous follicle. × 35.
FIG. 2.—The vestigial follicle shown in Fig. 1. × 350.
FIG. 3.—Rodent ulcer composed of spindle-shaped cells (Type A). × 85.
FIG. 4.—Part of the circumscribed spindle-celled tumour shown in Fig. 3. × 350.
FIG. 5.—Normal hair root from the scalp. × 50.
FIG. 6.—Part of Fig. 5 at a higher magnification to show the hair matrix cells. × 350.
FIG. 7.—A circumscribed rodent ulcer composed of Type B cells, with a well-marked palisade layer. × 85.
FIG. 8.—Part of the tumour illustrated in Fig. 7, showing the reticular arrangement. × 350.
FIG. 9.—Normal hair showing the resemblance of the outer root sheath to the rodent ulcer in Fig. 8. × 350.
FIG. 10.—Rodent ulcer showing whorls and parakeratin pearls. × 65.
FIG. 11.—The edge of a rodent ulcer showing rounded or circumscribed cell masses bounded by a palisade layer. × 30.
FIG. 12.—Part of Fig. 11 at a higher magnification. × 200.
FIG. 13.—The advancing edge of a rodent ulcer of infiltrative type, showing the fibrotic lacrimal gland below. × 30.
FIG. 14.—Columns of infiltrating cells from the tumour shown in Fig. 13. × 200.

Thackray.

Thackray.

11

12

13

14

Thackray.

In any investigation of this sort the issue tends to be confused by the number of variables involved. In addition to the histological features with which we are primarily concerned, there are variations of the technique of irradiation, in the original size of the lesion, and so on. If any conclusions are to be reached we must reduce these other variables to the simplest terms, and although on the cards the method of treatment was noted as surgical excision, or radiotherapy, the latter again as surface application of radium, insertion of radium, radon, and superficial or deep x-rays, these methods of treatment often being used in combination or successively, in the final analysis treatment was indicated as "surgery" or "radiotherapy." The results of treatment had also to be simplified, and the cases were classified eventually into A for cure following a single method of treatment, B where cure followed treatment of a recurrence, and C where the condition progressed despite treatment or there were numerous recurrences.

On the back of the card the histological features of the tumour were recorded.

The sections of the tumours in this series were studied and a certain number were noted as being probably of sweat-gland origin. The majority of the tumours were thought to be of pilar origin, and it was apparent that two main types of cell and arrangement could be made out in these pilar tumours. In one type the cells were spindle-shaped with relatively little cytoplasm (Fig. 3 and 4), and resembled those of the hair matrix (Fig. 5 and 6); tumours composed predominantly of such cells were noted on the cards as Type A. The other variety, Type B, was composed of rather smaller cells with relatively more cytoplasm, having a reticular arrangement, often with small clear intercellular spaces (Fig. 7 and 8), and resembling the cells of the outer sheath of the hair root (Fig. 9). In tumours composed of this type of cell whorls and parakeratin pearls were sometimes found (Fig. 10). These two types, between which there were many mixed and intermediate forms, were taken to represent differentiation of the tumour in the direction of different parts of the normal pilar unit.

The tumours were also divided up according to their habit of growth. The least invasive tumours, composed of rounded or at any rate circumscribed cell masses, usually having a well-marked palisade layer and basement membrane, and often with evidence of differentiation either in the direction of hair or sweat glands were classed as Group I (circumscribed) (Fig. 11 and 12). Other tumours were recognized as having an infiltrative habit of growth. The tumour cells, in columns or streaks, could be seen infiltrating the connective-tissue planes, and basement membranes were usually absent (Fig. 13 and 14); these were classed as Group III (infiltrative). A third intermediate group comprised those tumours which were predominantly circumscribed, but with evidence of invasiveness in places. Compression of the cell masses by newly formed fibrous tissue gave an effect likely to be confused with infiltration, and as this was most often seen following irradiation, sections of recurrences following such treatment were not classified in this way.

### ANALYSIS OF FINDINGS.

*Age, sex and site incidence.*

Before proceeding to the histological analysis of the cases we may note here the age and sex incidence of rodent ulcers, including for this purpose the older patients who had an insufficient period of follow-up for the main series. There were 148 males to 95 females, and their ages were distributed as shown in Table I.

TABLE I.—*Age Incidence of Rodent Ulcers.*

| Age-group. | Number. | Age-group. | Number. |
|---|---|---|---|
| 0–9 | 0 | 50–59 | 55 |
| 10–19 | 1 | 60–69 | 65 |
| 20–29 | 2 | 70–79 | 66 |
| 30–39 | 16 | 80+ | 9 |
| 40–49 | 29 | | |

As the 70–79 age-group contains fewer individuals than the two or three decades preceding it, it is obvious that the percentage incidence of the disease is at its highest in this group. This fact introduces a special difficulty into the assessment of the efficacy of treatment of rodent ulcers, for a considerable number of the patients will die from natural or other causes before an adequate follow-up period has elapsed, and are likely to be classed as "cured," when in fact the lesion might well have recurred had the patient lived longer. Insisting on a ten-year follow-up, as in this series, is bound to give a higher recurrence rate than those usually quoted.

The age noted on the cards was the patient's age at the beginning of treatment. Patients often have a hazy idea about when they first noticed the lesion and errors would certainly be introduced by attempts to date back their age to the first appearance of the growth.

The lesions were situated as shown in Table II. Out of the 210 ulcers, all but 20 were situated on the head and face, similar figures to those given by Wakeley and Childs (1949), who found 27 ulcers on the rest of the body as against 210 on the face or scalp. Of the 20 ulcers in the present series situated other than on the head, 2 failed to respond to treatment. The majority of ulcers occurred in the region of the eyes, nose, cheeks and forehead, with a smaller number of the scalp. More than one ulcer was present in 31 out of the 200 patients.

TABLE II.—*Site of Rodent Ulcers.*

| | | | |
|---|---|---|---|
| Cheek | 43 | Abdominal wall | 2 |
| Lip | 7 | Neck | 4 |
| Chin | 4 | Back and shoulders | 5 |
| Temple | 13 | Forearm | 1 |
| Scalp | 14 | Hand | 1 |
| Forehead | 20 | Breast | 3 |
| Eyelids | 45 | Leg | 1 |
| Nose | 34 | Groin | 1 |
| Ear | 10 | Vulva | 1 |
| | | Scrotum | 1 |
| | 190 | | 20 |

*Results of treatment.*

*Surgery.*—Primary surgical excision was the method of treatment used for 63 ulcers in this series. Of these, 12 subsequently recurred.

Histological examination of the excised ulcer is of value to the surgeon mainly as an indication as to whether removal has been complete. For this, serial sections through the whole lesion would obviously be necessary, but usually an opinion can be given on a section taken through the region where the tumour comes nearest to the edge of the specimen. The sections available did not always include the whole lesion, but where this was so a note was made as to whether excision appeared to be complete, incomplete or doubtful. Of the 12 cases which recurred, 8 were obviously incompletely excised, one appeared to have been completely removed from the section studied, and 3 were doubtful. Of the 51 successful excisions, 42 had been removed with an adequate margin of healthy tissue, one tumour reached to the limit of excision, and 3 came so near to the edge that the outcome seemed doubtful. In the remainder the sections available did not cover the whole lesion and no opinion as to completeness of removal could be given.

Of the 12 failures with surgery, one was cured by further excision, and 4 more by irradiation. Three of the remaining 7 died as a result of the disease, unchecked by further surgery or irradiation, 2 patients are still alive with the ulcer active, and the other 2 died of other causes with the ulcer uncured. The slow growth of some of these tumours is illustrated by one of these patients who lived 12 years after the incomplete excision of a rodent ulcer of the forehead. In all the completely failed cases the lesion was more than one inch in diameter when first seen or involved underlying bone or cartilage ; all these lesions were situated on the head or face.

*Radiotherapy.*—Radiotherapy was the primary method of treatment used for 121 of the ulcers in this series. Of these, 82 proved to be radiosensitive and did not recur, but 39 recurred within 10 years or persisted after the first course of treatment. Of these 39 ulcers, 19 were soon cured by further treatment, leaving 20 which proved radio-resistant.

It is proposed to compare the incidence of various histological features in the biopsies from the 82 lesions cured by a single course of radiotherapy with their incidence in the 20 radio-resistant ulcers. These figures incidentally introduced must not be considered as representative of the results of radiotherapy by modern methods, for it must be remembered that these cases were all followed for 10 years, the most recent treatment being therefore in 1938.

*Influence of size of lesion on prognosis.*

Before considering the histological analysis of these cases it is interesting to note the influence of the size of the lesion on the prognosis. The greatest measurement of the ulcer in inches was noted on the cards, and these sizes were then divided into two groups, large and small, taking one inch or more as the criterion of largeness. This boundary between large and small was placed quite arbitrarily and in fact there proved to be about twice as many small lesions as large.

Of the surgically excised lesions, the size had been noted in 51 cases, and of these 16 were large and 35 small. Of the tumours that did not recur after removal, 27 per cent were graded as large ; whereas of the tumours which did recur, 55 per cent were large.

In the group of cases cured by the first course of radiotherapy there were twice as many small ulcers as large ; in the radio-resistant group the ratio was more than reversed, there being three times as many large as small.

*Influence of type of lesion on prognosis.*

As has been described already, the lesions were classed on their histological appearance as being circumscribed (Group I), predominantly circumscribed, but with early infiltration present (Group II), or infiltrative (Group III) in their type of growth. It was hoped that this classification might give an indication of the malignancy of the tumours without introducing controversial problems concerning their origin.

It may be suggested that the infiltrative lesions are the large and advanced ones, and that any correlation we may note between type of growth and prognosis will merely be a repetition of what we saw in the preceding section. The whole series of surgical cases, including also those in which surgical excision was combined with radiotherapy in the first instance, was therefore grouped as to size of lesion and type of growth. The results are shown in Table III, omitting for the sake of clarity the Group II cases.

TABLE III.

| | Size of Lesion. | |
|---|---|---|
| Type of Growth. | Large. | Small. |
| Circumscribed | 9 | 24 |
| Infiltrative | 10 | 18 |

It will be seen from Table III that although the small lesions are more commonly of the circumscribed type, the large lesions are as often still circumscribed as infiltrative.

The correlation between the type of growth and the result of treatment can best be shown in the form of a table (Table IV).

TABLE IV.

| | | Cured. | Recurred. |
|---|---|---|---|
| Surgical cases | Group I | 29 | 0 |
| | Group II | 11 | 0 |
| | Group III | 11 | 12 |
| | | Success. | Failure. |
| Radiotherapy cases | Group I | 43 | 5 |
| | Group II | 12 | 0 |
| | Group III | 27 | 15 |

The figures in Table IV clearly show that the prognosis is far better with the circumscribed than with the infiltrative growths.

*Palisade layer.*

The next few histological features to be considered were assessed only for their bearing on the reaction of lesions to radiotherapy. Their incidence is therefore compared between the group of tumours which were cured by the first course of radiotherapy, and those which were uncured by more than one course of such treatment. To this latter group of cases were added 6 patients who were first treated by irradiation at some other hospital and whose recurrences proved radio-resistant here.

The cell masses which go to make up a rodent ulcer are often bounded peripherally by a layer of columnar cells, usually known as the palisade layer (Fig. 7 and 8). This layer was noted on the cards as being prominent, less well defined, or absent; only the first and last groups are considered here, and the correlation with the result of radiotherapy is shown in Table V.

TABLE V.

| | Success. | Failure. |
|---|---|---|
| Palisade layer well marked | 41 | 13 |
| Palisade layer absent | 11 | 9 |

Palisade formation is more often well-marked in the group which reacts favourably to irradiation than in the resistant lesions. There was only partial correlation between the presence of palisades and the circumscribed type of growth. The great majority of the lesions with no palisade formation apparent on section were of the infiltrative variety, whereas in the group with well-marked palisades 30 were circumscribed (Group I) and 19 infiltrative (Group III). Of these 19 infiltrative lesions, 10 were radio-resistant, so that it would suggest that the type of growth is of greater prognostic significance than the degree of palisade formation.

*Mitoses.*

Mitoses were noted as being " numerous " when they could be found without difficulty in the sections, often several in a field, as " present " when examples could be found easily but in smaller numbers, or as " absent." If we include also the surgical cases, then mitoses were numerous in 48, occasional or " present " in 32, and absent in 99. That is to say that 45 per cent of the lesions in this series showed mitoses, which were numerous in 27 per cent. The relation between the number of mitoses and the outcome of radiotherapy is shown in Table VI.

TABLE VI.

| Mitoses. | Success. | Failure. |
|---|---|---|
| Numerous | 18 | 6 |
| Present | 14 | 5 |
| Absent | 50 | 15 |

It clearly makes no odds to the outcome of radiotherapy whether the lesion shows many, few, or no mitoses.

*Pigment.*

The presence of melanin in the lesions was similarly noted as marked, slight or absent. The correlation is shown in Table VII.

TABLE VII.

| Pigmentation. | Success. | Failure. |
|---|---|---|
| Present | 5 | 0 |
| Slight | 2 | 1 |
| Absent | 75 | 25 |

Pigmentation was thus present in only 8 out of the 108 cases, and the figures are too small for the assessment of prognostic significance: at any rate all the cases in which pigmentation was marked were cured by the first course of irradiation, so that this would not appear to be a contraindication to such treatment. Pigmentation only seems to be at all well marked in the better differentiated lesions. In 2 tumours in the series pigmentation was very prominent; both were diagnosed clinically as malignant melanoma and were widely excised together with the regional lymph glands, and both patients were cured. This possibility of clinical confusion with radio-resistant tumours of the melanoma group results in the majority of pigmented rodents being treated surgically.

*Mucus and cysts.*

Table VIII shows the relation between mucoid degeneration and cyst formation in the tumours and their reaction to radiotherapy.

TABLE VIII.

| | Result of Irradiation. | |
|---|---|---|
| | Success. | Failure. |
| Mucus + | 32 | 12 |
| Mucus — | 46 | 14 |
| Large cysts | 9 | 2 |
| Small cysts | 12 | 3 |
| No cysts | 57 | 21 |

The cysts, where present, contained mucoid material. Mucus, when present in the absence of cysts, was intercellular, around the periphery of the cell masses, or in the stroma. It is clear that no prognostic significance can be attached to the presence of mucus or cysts in the lesions.

*Cell type.*

There were only 11 tumours in the series that were thought to have originated in sweat glands. Five of these, all circumscribed lesions, were excised without recurrence. The other 6 were treated by radiotherapy; one recurred but was cured by subsequent excision, 4 were cured by the first course of treatment and the sixth case, first treated 20 years ago, is still active despite repeated radiotherapy and surgical excision. There are clearly not enough cases of this type for statistically significant conclusions to be drawn, but the sudoriparous growths appear to behave much like the general run of rodent ulcers.

TABLE IX.

| | Type A. | Type B. |
|---|---|---|
| Irradiated successfully | 28 | 28 |
| Qualified success | 5 | 9 |
| Unsuccessful irradiation | 11 | 6 |
| Surgically excised | 21 | 19 |
| Total | 65 | 62 |

The pilar tumours were divided into two groups depending on whether the larger spindle-shaped cells resembling those of the hair matrix predominated, or the smaller cells having a reticular arrangement like those of the hair sheath. The results of irradiating these two types are shown in Table IX, designating the two types A and B respectively.

It appears from Table IX that the two types are equally common and that they do not differ significantly in their response to irradiation. It is important to remember that these are not clear-cut types, but represent tendencies towards different parts of the pilar unit, and some areas of both types could be found in most tumours.

*Basal squamous carcinoma.*

Of the 121 ulcers treated by radiotherapy only 12 were placed in this group; and since of these 12 no less than 6 were uncured, there would seem to be some evidence to support their relative radio-resistance. The numbers are still small, however, and a further investigation is planned into the reaction to irradiation of the larger numbers of these growths seen in recent years.

### CONCLUSIONS.

The prognosis for the patient following the surgical removal of a malignant tumour, for example a carcinoma of the breast, depends on whether it has already extended directly or by metastasis beyond the region excised. If the primary tumour only is available for examination, the likelihood of metastasis having occurred can be gauged from the histological grade of malignancy of the growth so that the grading alone is of considerable prognostic significance.

With a surgically excised rodent ulcer the problem is simpler. The question of metastasis does not arise, and it is only necessary to be sure that the margin of the excised tissue is free from growth. This can be done by cutting serial sections of the excised tissue, which is well worth while if there is any doubt. If serial sections are not prepared, then the nature of the growth itself gives a fair indication, for tumours of the circumscribed type, as we have called it, are relatively compact and their extent is apparent from the surface, whereas infiltrative growths extend beyond their apparent limits.

When considering the results of radiotherapy it is important to distinguish clearly between radio-sensitivity and radio-curability. A tumour which melts away when irradiated may often recur, whereas the tumour which can be destroyed curing the patient, may show no dramatic immediate response. In this investigation the ten-year cure is the criterion of successful treatment, so that radio-curability is all we are concerned with. It is well known that the histological grade of malignancy as applied to carcinomas of the skin, say, or breast, has some bearing on the outcome of radiotherapy—those of low-grade malignancy often being curable, those of high malignancy usually being sensitive, but recurring. In carcinomas of the breast such features as the degree of tubule formation, variability in size and staining of the nuclei and the frequency of mitoses are all significant, and are taken into account in determining the grade. The results of the present investigation have been largely negative, for most of the histological features of rodent ulcers considered have been shown to be without significance in assessing the probable outcome of treatment. Thus neither mucus formation, the presence of cysts, pigmentation, nor even the number of mitoses seem to have any bearing on the question.

The only method of dividing up the tumours which seemed to bear any relation to the prognosis was that based on the habit of growth, into circumscribed or infiltrative, and the fact that an intermediate group was necessary indicates the difficulty in making this distinction. Grading as practised in connection with carcinomas of the breast and elsewhere is an indication of the degree of malignancy of malignant tumours ; the term " Group " has been used here rather than " Grade " in view of the suspicion that many of the Group I tumours are probably in actual fact benign. The classification of rodent ulcers into pilar and sudoriparous varieties is difficult, and there is no evidence that they differ in their behaviour.

### SUMMARY.

1. A series of 850 rodent ulcers has been examined ; 200 cases that had been followed up for 10 years or more have been studied in detail.

2. It was concluded that rodent ulcers originate in hair follicles, in sweat glands, or in downgrowths of the surface epithelium tending towards the reproduction of these structures.

3. The better differentiated rodent ulcers can be related either to hair follicles or to sweat glands, but this distinction is often impossible with the less differentiated tumours, and in any case seems to have no effect on the prognosis.

4. Of the other histological features studied, only the extent to which growth is infiltrative seemed to have a direct bearing on the prognosis and prospect of cure by radiotherapy.

My thanks are due to the surgeons of the Middlesex Hospital and to Professor B. W. Windeyer of the Meyerstein Institute of Radiotherapy, for allowing me to study their cases, and to Miss J. Chambers of the Follow-up Department for tracing the patients.

This investigation was carried out during the tenure of the Gillson Scholarship of the Society of Apothecaries in 1948.

### REFERENCES.

Darier, J., and Ferrand, M.—(1922) *Ann. Derm. Syph., Paris*, **3**, 385.
Foot, N. C.—(1947) *Amer. J. Path.*, **23**, 1.
Haythorn, S. R.—(1931) *Amer. J. Cancer*, **15**, 1969.
Krompecher, E.—(1900) *Beitr. path. Anat.*, **28**, 1.
Wakeley, C., and Childs, P.—(1949) *Brit. med. J.*, i, 737.